# Supplementary material for: Biodegradable Nanoparticles Loaded with Levodopa and Curcumin for Treatment of Parkinson’s Disease
Source: Molecules. 2022 Apr 28;27(9):2811. doi: 10.3390/molecules27092811 (PMC9101601; doi:10.3390/molecules27092811)
Supplement: Supplementary file 1 [file molecules-27-02811-s001.zip › molecules-1657391-supplementary.pdf]

Supplementary Materials

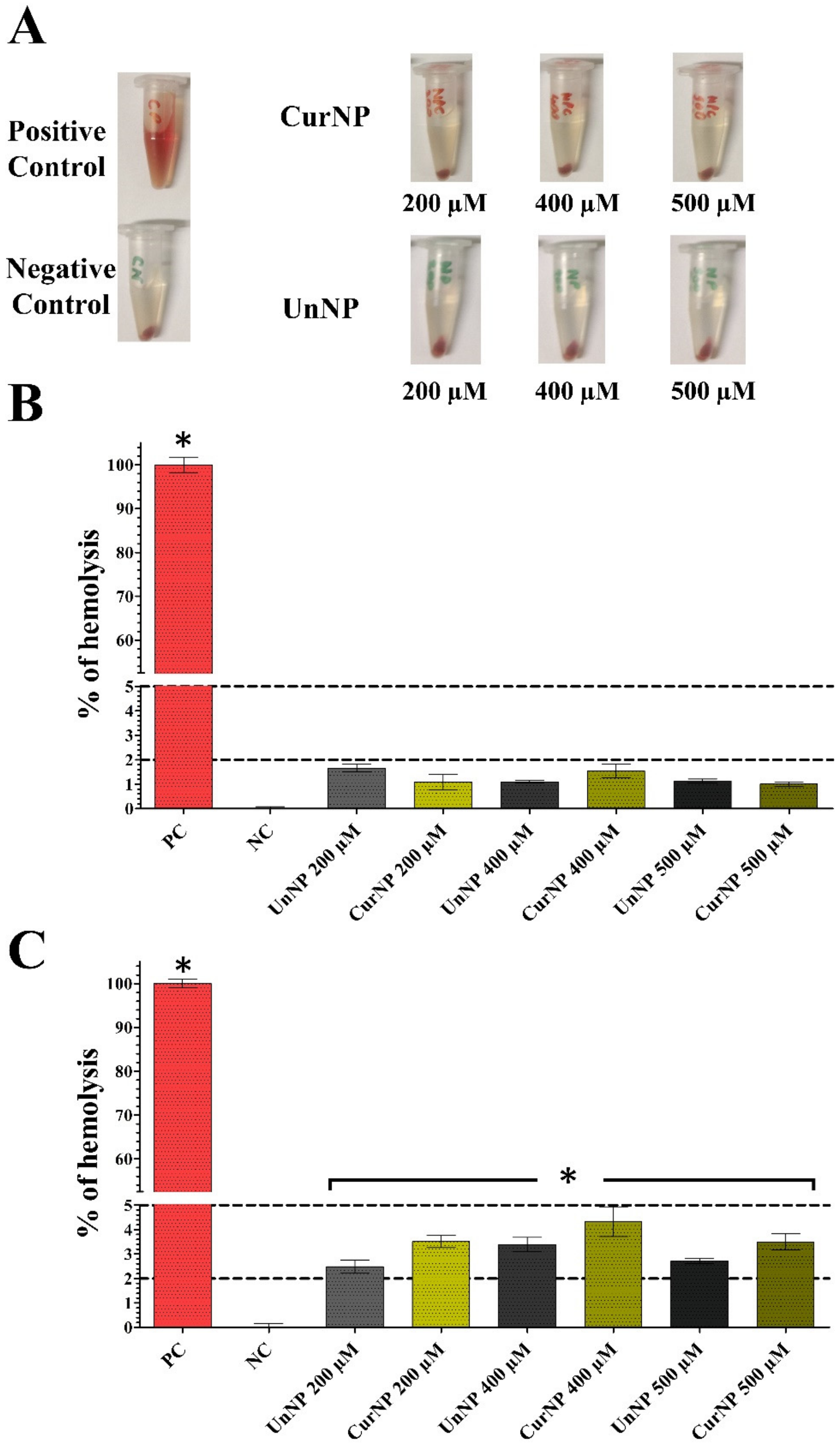

Figure S1. (A) Hemolysis assay microtubes after incubation with nanoparticles suspensions in different concentrations (200, 400 and 500µM) for 1 hour and centrifugation at 1500G for 5 minutes Positive Control, distilled water (PC), Negative Control, saline (NC), Unloaded Nanoparticles (UnNPs), Curcumin-loaded Nanoparticles (CurNPs). Curcumin-loaded Nanoparticles (B) and (C) The results presented are mean ± standard error of the hemolysis rate of samples 1 and 2, respectively. Dotted lines are the thresholds of 5% (slightly hemolytic) and 2% (non-hemolytic) hemolysis rate and * $p < 0.05$; n.s. $p > 0.05$ when compared with the negative control.

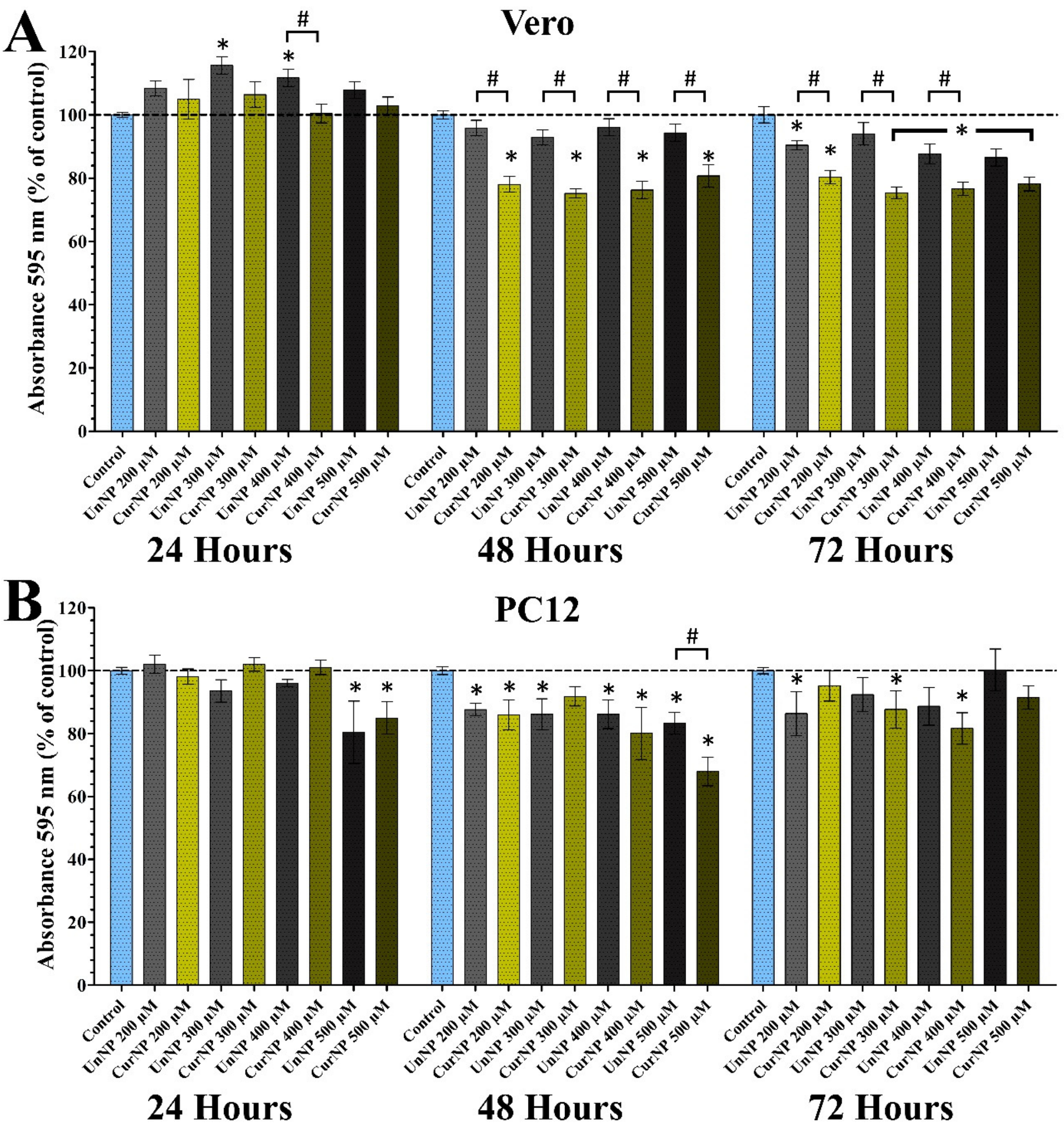

Figure S2. MTT assays of Vero and differentiated PC12 cells after 24, 48, and 72 hours of incubation with the nanoparticles' suspensions in different concentrations. Absorbance was determined in a spectrophotometer at a wavelength of 595nm, and values are presented as mean ± standard error of the % of the control group (* and # $p < 0.05$; n.s. $p > 0.05$). (A) Vero cells treated with Curcumin-loaded nanoparticles (CurNP) and unloaded nanoparticles (UnNP) suspensions in a concentration of 200, 300, 400, and 500µM. (B) differentiated PC12 cells treated with CurNP and UnNP suspensions in concentrations of 200, 300, 400, and 500µM.

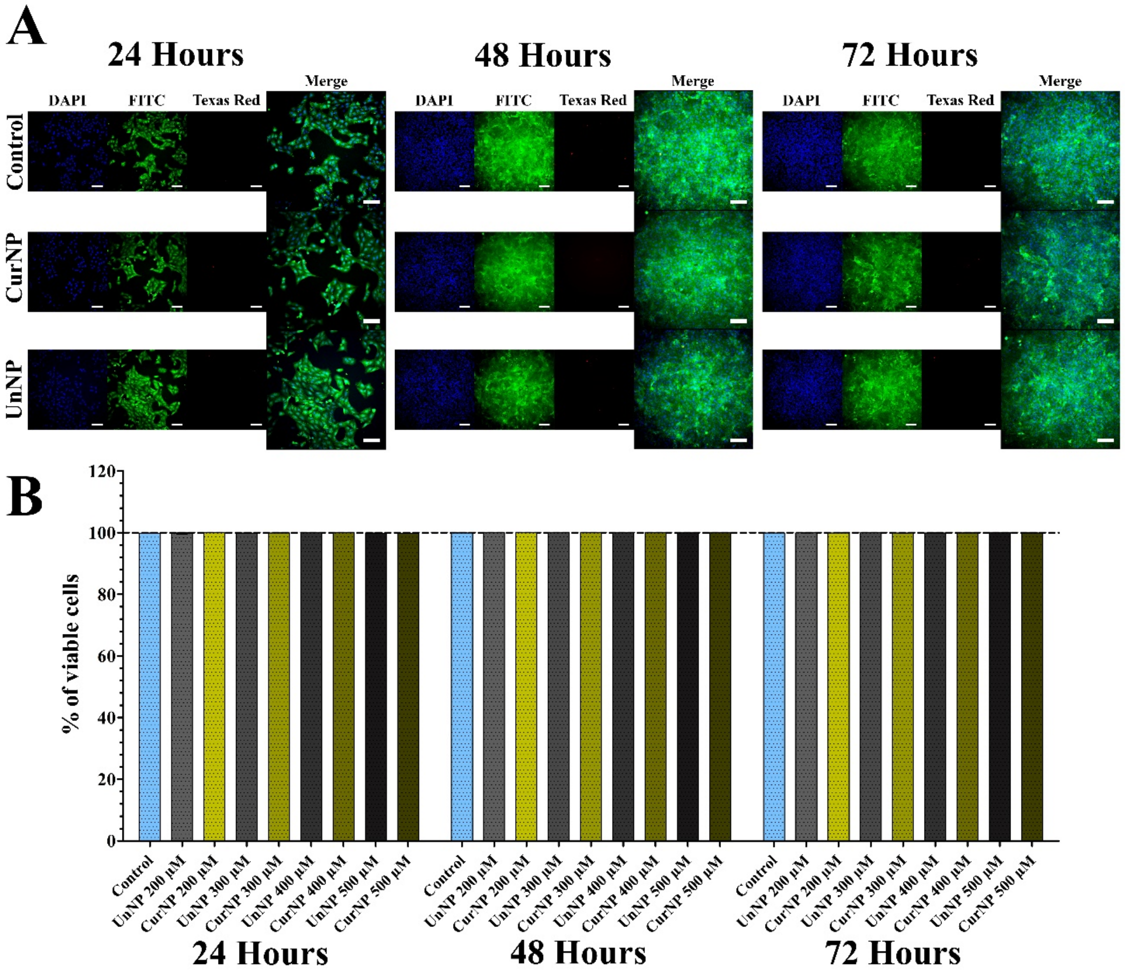

Figure S3. LIVE/DEAD viability assays of differentiated Vero cells after 24, 48, and 72 hours of incubation with the nanoparticles' suspensions in different concentrations. (A) Representative images of control, Curcumin-loaded Nanoparticles (CurNP) 500µM and Unloaded Nanoparticles (UnNP) 500µM. Cells nuclei were stained with Hoechst 33342 and observed with the DAPI channel, live cells were stained with calcein and observed in the FITC channel, dead cells were stained with ethidium homodimer-1 and observed in the Texas Red channel. (Scale bar 100µm). (B) CurNP and UnNP suspensions in concentration of 200, 300, 400 and 500µM, Values were presented as mean ± standard error of the % of viable cells (* $p < 0.05$; n.s. $p > 0.05$).

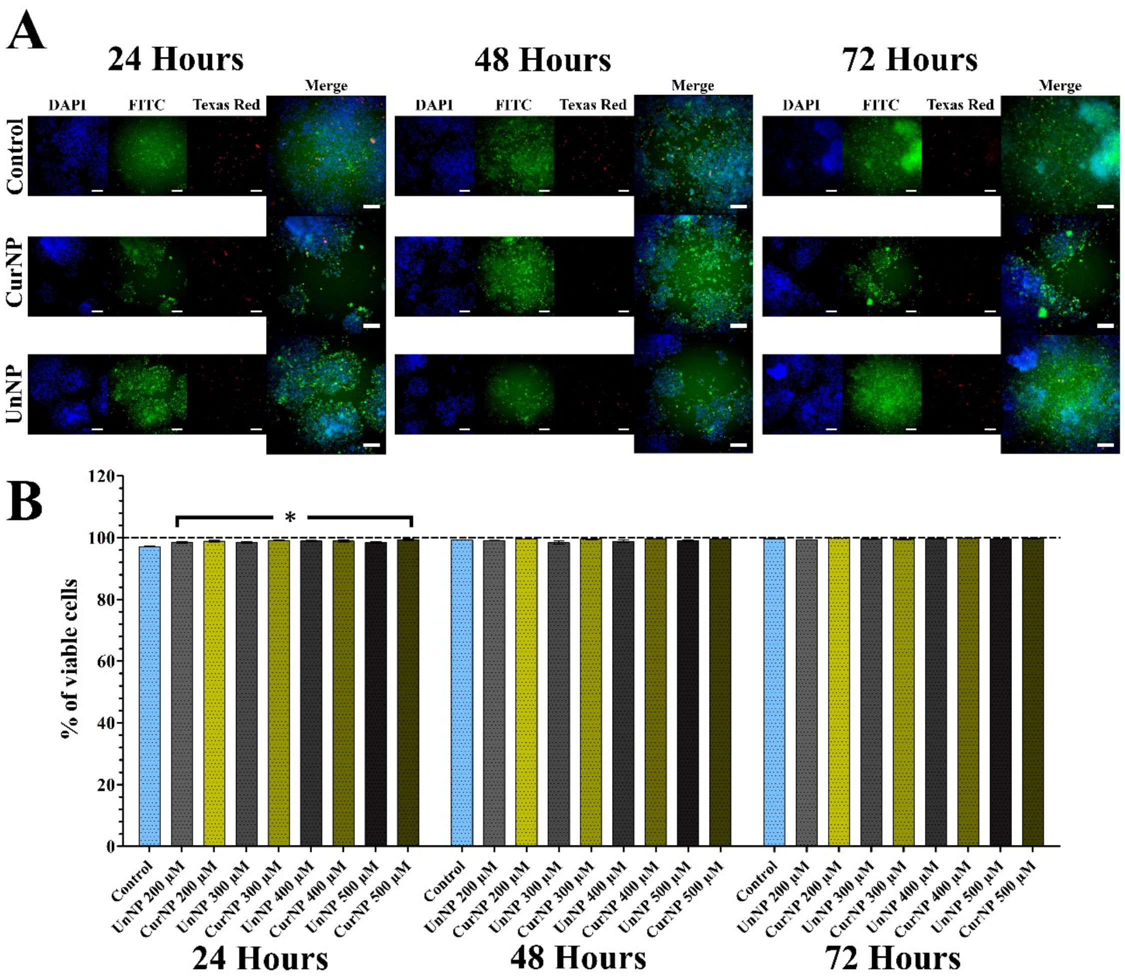

Figure S4. LIVE/DEAD viability assays of differentiated PC12 cells after 24, 48, and 72 hours of incubation with the nanoparticles' suspensions in different concentrations. (A) Representative images of control, Curcumin-loaded Nanoparticles (CurNP) 500µM and Unloaded Nanoparticles (UnNP) 500µM. Cells nuclei were stained with Hoechst 33342 and observed with the DAPI channel, live cells were stained with calcein and observed in the FITC channel, dead cells were stained with ethidium homodimer-1 and observed in the Texas Red channel. (Scale bar 100µm). (B) CurNP and UnNP suspensions in concentration of 200, 300, 400 and 500µM, Values were presented as mean ± standard error of the % of viable cells (* $p < 0.05$; n.s. $p > 0.05$).
